# Cinnamaldehyde/β-Cyclodextrin Inclusion Complex Enhances Physicochemical and Antioxidant Properties of Edible Orally Disintegrating Film

**DOI:** 10.3390/foods15081410

**Published:** 2026-04-17

**Authors:** Yaxin Zhou, Yachao Tian, Haojie Sha, Caihua Liu, Shutao Guo, Zhongjiang Wang

**Affiliations:** 1 State Key Laboratory of Medicinal Chemical Biology, Key Laboratory of Functional Polymer Materials of Ministry of Education, Institute of Polymer Chemistry, College of Chemistry, Nankai University, Tianjin 300071, China; zhouyaxinarya@163.com; 2 College of Food Science, Northeast Agricultural University, Harbin 150030, China; tianyachaopaper@163.com (Y.T.); shahaojiede@163.com (H.S.); lchname@126.com (C.L.)

**Keywords:** cinnamaldehyde/β-cyclodextrin inclusion complex, soy protein isolate, orally disintegrating film, physicochemical properties, antioxidant stability

## Abstract

Despite the growing interest in orally disintegrating films (ODFs), developing soy protein isolate (SPI)-based ODFs with both rapid disintegration and high functional stability remains a challenge. This study developed a novel SPI-based ODF incorporated with a cinnamaldehyde/β-cyclodextrin (CA/β-CD) inclusion complex at varying concentrations (5–20%, *w*/*w*) to address this gap. The control ODF exhibited poor structural order, a slow disintegration rate, and weak antioxidant activity. The incorporation of an appropriate amount of CA/β-CD inclusion complex (10–15%) significantly improved the comprehensive properties of the ODFs. The inclusion complex facilitated the formation of an orderly, continuous network structure, leading to a substantial enhancement in tensile strength (TS), elongation at break (EAB), disintegration rate, thermal stability, and sustained antioxidant activity. An excessive inclusion complex concentration (20%) induced agglomeration, compromising the structural integrity and functionality of the ODF. FTIR and secondary structure analyses revealed that the enhanced hydrogen bonding between the CA/β-CD inclusion complex and the SPI matrix promoted the transformation of disordered protein structures into ordered conformations (β-sheets and α-helices). This structural ordering is the core mechanism driving the improved macroscopic physicochemical and functional properties of the ODFs. This study confirms that CA/β-CD inclusion complexes can enhance the performance of SPI-based ODFs and provide a highly promising delivery system for hydrophobic bioactive substances.

## 1. Introduction

Orally disintegrating films (ODFs) are novel delivery carriers for drugs and active substances, which usually look like a small stamp or mint paper [1]. Even without the aid of water, ODFs can rapidly disintegrate and disperse in the oral cavity and release the loaded active substances [2]. ODFs are categorized into fast-disintegration films, which dissolve in less than 60 s, and slow-disintegration films, which take more than 60 s [2,3]. Films with a faster disintegration rate enable the more rapid release and absorption of the loaded active ingredients, thereby enhancing their efficacy [4]. Therefore, it has become the focus in the field of functional foods to identify raw materials for preparing ODFs with both safety and rapid disintegration properties.

Numerous natural materials, including pregelatinized cassava starch [3], hydroxypropyl methylcellulose [5], pectin [6], hyaluronic acid [7], and soy protein isolate (SPI) [2], have been validated for developing ODFs. SPI is a natural polymer derived from soybeans, which includes all essential amino acids for humans, has a PDCAAS of 1.0, and aids in digestion and absorption [8]. Compared with polysaccharide-based raw materials, SPI offers superior nutritional value, rendering it a promising candidate for ODF fabrication [2]. Owing to its biocompatibility and biodegradability, SPI holds substantial potential for application in active component delivery systems [9,10].

A significant challenge in ODF development is oxidative degradation, which compromises product quality and raises safety concerns [11,12]. Cinnamaldehyde (CA) is a natural bioactive substance with various physiological activities, such as antioxidant, antibacterial, and anti-inflammatory effects [13]. Studies further indicate its potential in promoting gingival health and preventing periodontitis [14,15]. However, the practical application of CA is limited by its poor aqueous solubility, high volatility, and instability [16,17]. If CA is directly added to the ODF matrix, its efficacy is seriously affected due to uneven distribution and volatilization during processing. Research has demonstrated that encapsulation technology can efficiently enhance the stability of antioxidant bioactive substances and facilitate their sustained release [11]. β-cyclodextrin (β-CD) is a cyclic oligosaccharide produced by the catalytic hydrolysis and cyclization of starch via cyclodextrin glucosyltransferase [13]. Its structure consists of seven D-glucose units connected by α-1,4-glycosidic bonds, with the units arranged into a hollow conical shape featuring a hydrophobic internal cavity and a hydrophilic exterior [18]. This unique structure enables β-CD to form inclusion complexes with lipophilic or volatile molecules, improving solubility, masking odors, and protecting bioactive compounds [19]. Previous research has confirmed that β-CD encapsulation effectively preserves CA bioactivity [13,17]. A prior study indicated that the incorporation of catechin/β-CD inclusion complexes into pregelatinized starch-based ODFs could significantly prolong the onset time of catechin in ODFs, a result attributed to the encapsulation effect of β-CD, which efficiently protects bioactive components and achieves a sustained-release effect [12]. Inspired by these findings, we hypothesize that the addition of CA/β-CD inclusion complexes into SPI matrices can effectively enhance the antioxidant performance of ODFs. To the best of our knowledge, limited studies have addressed the efficacy enhancement of SPI-based ODFs by incorporating CA/β-CD inclusion complexes into the SPI film-forming matrix. Currently, a significant knowledge gap exists regarding how the inclusion complexes influence the protein secondary structure and macroscopic network of SPI-based films, as most prior studies have focused on polysaccharide-based matrices [3,12]. CA/β-CD inclusion complexes are uniquely suited for this application, as β-CD not only protects the highly volatile CA but also introduces abundant hydrophilic hydroxyl groups that can interact with the SPI protein chains.

This study initially developed a CA/β-CD inclusion complex and thoroughly examined its impact on the microstructure, mechanical properties, hydrophilicity, disintegration behavior, antioxidant capacity, and thermal stability of SPI-based ODFs. This work provides a viable strategy for fabricating high-performance functional ODFs and offers important insights into developing safe and efficient delivery systems for CA and other bioactive compounds.

## 2. Materials and Methods

### 2.1. Materials

CA (purity > 98%) was obtained from Sigma-Aldrich (Shanghai, China), while β-CD (purity 99%) was sourced from Shanghai Yuanye Biotechnology Co., Ltd. (Shanghai, China). SPI (protein purity ≥ 90%, molecular weight = 150–360 kDa, gel value ≥ 170 g, moisture content ≤ 7%) was generously supplied by Shandong Yu Wang Ecology Food Industry Co., Ltd. (Dezhou, China). The chemical structures of CA and β-CD are provided in Figure S1 (Supplementary Materials). All other chemicals utilized in the experiments were of analytical grade.

### 2.2. Preparation of CA/β-CD Inclusion Complex

The CA/β-CD inclusion complex was prepared by the saturated aqueous solution method, and the specific method was adapted with slight modifications from our previous study [13]. A saturated solution was prepared by dissolving β-CD powder in deionized water. Then, CA was added to the β-CD solution drop by drop to form a mixed solution with a molar ratio of β-CD to CA of 1:1.5. Under dark conditions, the mixture was magnetically stirred in a 40 °C water bath for 36 h. The final product was obtained by freeze-drying the solution and was stored under desiccation for subsequent analysis.

### 2.3. ODF Preparation

Following the method reported by Jiang et al. (2021) [11], SPI-based ODFs were prepared by the solution casting method. Briefly, 1 g of SPI was dispersed in distilled water to prepare a 1% (*w*/*v*) SPI dispersion. Subsequently, CA/β-CD inclusion complexes were incorporated at concentrations of 5%, 10%, 15%, and 20% (*w*/*w*, based on the mass of SPI), respectively. This concentration range was established based on preliminary experiments. Below 5%, the improvement in ODF performance was negligible, while above 20%, obvious aggregation occurred. The mixtures were stirred at 45 °C for 20 min to ensure homogeneity. Each solution was then mixed with sorbitol (30% *w*/*w*, based on SPI mass) and stirred for an additional 15 min to obtain the film-forming solution. A volume of 5 mL of this solution was cast into a mold and dried at 40 °C for 10 h, after which the ODFs were peeled off. An ODF prepared without the inclusion complexes served as the control. The ODFs containing different amounts of the CA/β-CD inclusion complex were designated as CCS-1 (5% *w*/*w*), CCS-2 (10% *w*/*w*), CCS-3 (15% *w*/*w*), and CCS-4 (20% *w*/*w*), with all percentages based on SPI mass. All ODF samples were stored in a desiccator at 25 °C and 53% relative humidity for at least 48 h prior to further analysis to ensure equilibration.

### 2.4. Fourier Transform Infrared (FTIR) Spectroscopy Analysis

FTIR spectroscopy was performed using a Bruker Tensor 27 spectrometer (Bruker Optik GmbH, Ettlingen, Germany). Before detection, all samples were dried under vacuum for 2 h to remove water. The spectra were then recorded in the range of 4000 to 500 cm^−1^ with 16 scans under a nitrogen atmosphere.

### 2.5. SEM

The microstructure of ODFs was observed with a Hitachi TM3000 benchtop SEM (Hitachi High-Tech Corporation, Tokyo, Japan) following the methodology described by Sha et al. (2022) [12]. All samples were gold sputter-coated before observation and imaged at 500×, 1000×, and 2000× magnifications to analyze surface morphology.

### 2.6. Thickness

The thickness of each ODF was determined using a digital micrometer at five randomly selected points on the ODF. The ODF thickness is represented by the calculated mean and standard deviation of these measurements.

### 2.7. Moisture Content

Moisture content was determined according to the method of Tian et al. (2024) [2] with modifications. Pre-weighed ODF samples were dried at 105 °C for 12 h to constant weight. Moisture content was calculated as

(1) $$Moisture\;Content\;(\%)=\frac{W_{1}-W_{2}}{W_{1}}\times 100$$

where *W*_1_ and *W*_2_ are the weights before and after drying. All measurements were performed in triplicate.

### 2.8. Water Contact Angle

The static water contact angles of the ODFs were determined using a KRÜSS DSA 25 drop shape analyzer (KRÜSS GmbH, Hamburg, Germany) at room temperature. A 3 μL deionized water droplet was deposited onto the ODF surface, and the contact angle was measured immediately. The average value was calculated from tests conducted at a minimum of five random locations per sample.

### 2.9. Surface pH

Surface pH was measured following the method reported by dos Santos Garcia et al. (2020) [20]. ODF samples were moistened with 1 mL of PBS (pH 6.8), and a pH electrode was placed on the sample surface after 30 s. Readings were taken after 1 min. All measurements were performed in triplicate.

### 2.10. Mucoadhesiveness

The oral mucoadhesive strength of the ODFs was assessed using a TA.XT Plus texture analyzer (Stable Micro Systems Ltd., Godalming, Surrey, UK), with modifications to the method reported by Alaei et al. (2021) [21]. ODF samples (1 cm × 1 cm) were affixed to the cylindrical probe and brought into contact with a mucin-mimetic substrate (4% gellan gum + 2% glycerol) wetted with 500 µL PBS (pH 6.8). A contact force of 1.0 N was applied for 15 s to ensure intimate contact between the ODF and the mucin-mimetic substrate, followed by separation at a speed of 0.2 mm/s. The maximum force required for detachment was recorded as the mucoadhesive strength. All measurements were performed in triplicate, and results were expressed as mean ± standard deviation (SD).

### 2.11. Mechanical Properties

The mechanical properties of the ODFs, including tensile strength (TS) and elongation at break (EAB), were evaluated according to the method described by Tian et al. (2024) [2] using an autotensile tester (Stable Micro Systems). ODF strips (40 × 10 mm) were stretched at 40 mm/min until fracture. TS and EAB were calculated as

(2) $$TS\;(MPa)=\frac{F}{A}$$

(3) $$EAB\;(\%)=\frac{\Delta L}{L}$$

where *F* (N) represents the peak force at ODF fracture, *A* denotes the ODF’s cross-sectional area, Δ*L* is the elongation at break, and *L* is the initial length.

### 2.12. Disintegration Time

The in vitro disintegration time of the ODFs was determined with minor modifications to the method reported by Tian et al. (2024) [2] with modifications. ODF pieces (15 × 15 mm) were fixed on a frame, and 200 µL of distilled water was dropped onto the surface. The time taken for the complete disintegration of the film was recorded. All measurements were performed in triplicate.

### 2.13. Thermal Properties

Differential scanning calorimetry (DSC) was employed to assess thermal stability following a procedure adapted from Sha et al. (2022) [3] and aligned with standard methodology. A Mettler Toledo DSC 3+ (Mettler-Toledo AG, Greifensee, Switzerland) was used for the analysis. DSC analysis was performed using standard aluminum crucibles (non-hermetic) under a nitrogen flow of 20 mL/min. Only the first heating scan was recorded from 20 to 120 °C at 10 °C/min. All films were preconditioned at 25 °C and 53% RH for 48 h before testing to ensure consistent moisture content. Enthalpy change (ΔH) was calculated by integrating the endothermic region from 20 to 120 °C using the instrument’s thermal analysis software. The ΔH was calculated by integrating the heat flow curve from 20 °C to 120 °C using the Mettler Toledo STARe V16.10 software, following the equation

(4) $$\Delta H=\frac{1}{m}\int_{20^{\circ }C}^{120^{\circ }C}(q(T)-q_{baseline}(T))dT$$

where *m* is the mass of the ODF sample, *q*(*T*) is the measured heat flow, and $q_{baseline}(T)$ is the fitted baseline heat flow.

### 2.14. Antioxidant Activity

ODF antioxidant activity was assessed via the DPPH method. Briefly, ODF samples (20 mg) were shaken in 5 mL of methanol for 2 h in the dark. Following this, 2 mL of the resulting extract was reacted with 2 mL of a freshly prepared 0.1 mM DPPH methanolic solution. The mixture was kept in the dark at room temperature for 30 min before its absorbance was measured at 517 nm on a UV-Vis spectrophotometer. Measurements included a methanol blank and a sample-free DPPH control; all measurements were performed in triplicate.

### 2.15. Statistical Analysis

Data analysis was performed with SPSS Statistics 20 software (IBM Crop., Armonk, NY, USA). Significant differences (*p* < 0.05) between groups for each parameter were indicated by distinct letters. All data are presented as mean ± standard deviation (SD).

## 3. Results and Discussion

### 3.1. FTIR Analysis of CA/β-CD Inclusion Complex

**Figure 1:**
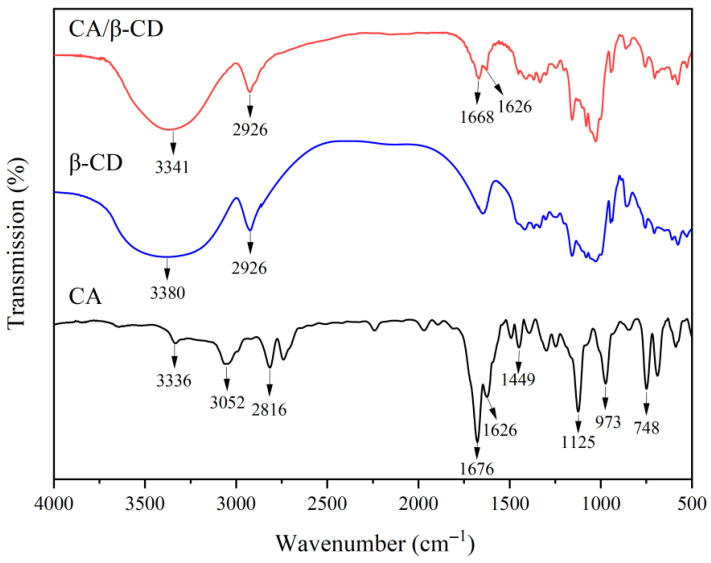
FTIR spectra of CA, β-CD, and the CA/β-CD inclusion complex.

The formation of inclusion complexes can be determined by comparing the infrared spectra of the host (β-CD), guest (CA), and the inclusion complex and observing changes in the characteristic peaks of functional groups [12]. Thus, FTIR was employed to verify the formation of the CA/β-CD inclusion complex. The infrared spectra of CA, β-CD and CA/β-CD inclusion complex are shown in Figure 1. The results of structural characterization showed that the characteristic peaks of β-CD at 3341 cm^−1^ (O-H stretching vibration of hydroxyl groups) and 2926 cm^−1^ (C-H stretching vibration) remained in the CA/β-CD inclusion complex, but the peak shapes changed. The characteristic peaks of CA at 3336 cm^−1^ (O-H stretching vibration), 1676 cm^−1^ (aldehyde C=O stretching vibration), 1449 cm^−1^ (C=C skeletal vibration), and 748 cm^−1^ (fingerprint region characteristic peak) exhibited significant changes in the spectrum of the inclusion complex. Specifically, the aldehyde characteristic peak of CA shifted from 1676 cm^−1^ to 1668 cm^−1^, while CA characteristic peaks at 3052 cm^−1^, 2816 cm^−1^, 1449 cm^−1^, and 748 cm^−1^ were notably weakened or disappeared. The redshift of the aldehyde C=O peak indicates a reduction in the C=O bond force constant, suggesting that the aldehyde group of CA is embedded within the hydrophobic cavity of β-CD, thereby weakening the conjugation effect [13,22]. Similar spectral changes have been reported for other volatile compounds encapsulated by β-CD, such as catechin [12] and essential oils [17], confirming that hydrophobic interactions and hydrogen bonding are the primary driving forces for complex formation.

### 3.2. SEM Analysis of ODFs

**Figure 2:**
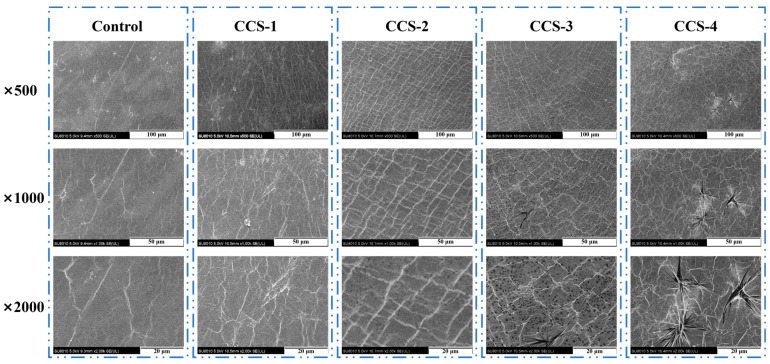
SEM images of ODF samples.

Figure 2 shows SEM images (500×, 1000×, and 2000× magnification) of different ODF samples. It can be seen from the figure that the morphological evolution of ODFs was highly correlated with the amount of the CA/β-CD inclusion complex added. The control ODF surfaces at all magnifications were predominantly flat with scattered, irregular cracks. This behavior can be explained by the fact that the pure SPI molecular chains rely solely on intrinsic hydrogen bonding and hydrophobic interactions, leading to uneven shrinkage stress during drying and molding, and thus resulting in poor structural compactness and a disordered microstructure. For the CCS-1 group, surface cracks were reduced, and slight fibrous textures appeared. This is due to the inclusion complex with low addition filling the gap between SPI molecular chains, relieving shrinkage stress and making the structure more uniform. However, the dispersion of the inclusion complex was limited at this stage, and an ordered skeleton had not yet formed. The CCS-2 ODFs exhibited a clear, regular, and continuous mesh-like structure at all magnifications, with cracks essentially absent. This indicates that a moderate amount of the inclusion complex, filling the interstices between SPI molecules, could align cooperatively with the protein chains, facilitating the construction of a uniform network support skeleton. A similar view was reported by Tian et al. (2024) [2], who suggested that small molecules like β-CD can fill the gaps between SPI chains, reducing pore formation. For the CCS-3 ODF, the network structure became roughened, with the local appearance of tiny pores and texture damage. With a further increase in inclusion complex content, the CCS-4 ODF surface showed numerous acicular aggregates accompanied by increased cracks and pores. Crucially, these morphological differences directly correlate with mechanical properties. The well-defined network in CCS-2 provides efficient stress transfer and load distribution, leading to peak tensile strength and elongation, whereas the aggregates and cracks in CCS-4 act as stress concentrators that initiate premature failure under tension. This is likely because the high-dosage inclusion complex exceeds the loading capacity of the SPI matrix. The excess inclusion complex was expelled from the SPI matrix, forming acicular aggregated phases, while these aggregates exacerbated stress concentration during drying, ultimately damaging the ODF structure. A previous study obtained a similar result to ours [17]. They found that the structure of starch-based films was dense and uniform by adding an appropriate amount of CA/β-CD inclusion complex, while excessive inclusion complex would be excluded from the substrate of the films, destroying the continuous network structure of the films. These morphological observations confirm that the dosage of the CA/β-CD inclusion complex is a key regulator of ODF microstructure and performance, as moderate addition optimizes the structure while excessive addition impairs it.

### 3.3. Thickness Analysis of ODFs

**Figure 3:**
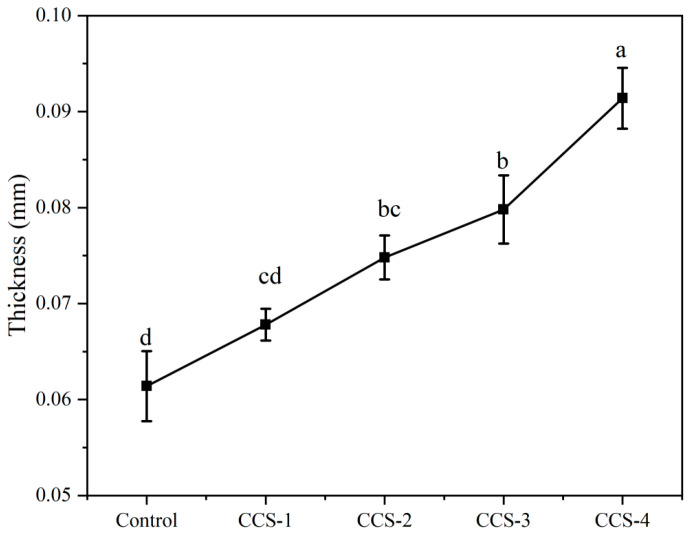
Thickness of ODF samples. Data are presented as mean ± SD; different letters indicate significant differences (*p* < 0.05) by one-way ANOVA with Tukey’s post hoc test.

Thickness is a critical parameter that directly determines the physicochemical and functional performance of ODFs [2]. The thickness variation of different ODF samples is shown in Figure 3. As illustrated in the figure, all prepared ODFs exhibited a thickness of less than 0.1 mm, with the control group showing the smallest thickness (0.061 mm). With the increase in the content of the CA/β-CD inclusion complex, the thickness of the ODFs increased gradually. This observation, consistent with prior literature, has been explained through two main mechanisms [2,3,17]. One interpretation associates the thickening with the overall increase in solid content of the film-forming solution [3]. Another explanation suggests that the inclusion complex acts as a filler within the SPI matrix, occupying spaces between the protein chains and mitigating volumetric contraction during drying, which results in a final film with greater thickness [2,22].

### 3.4. Moisture Content Analysis of ODFs

**Figure 4:**
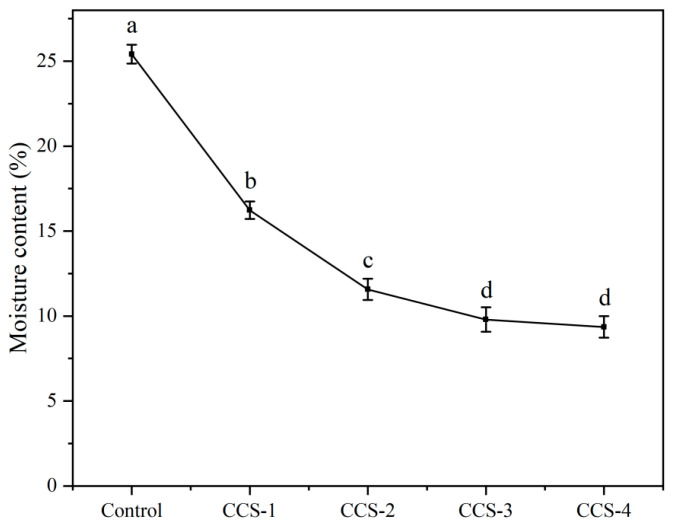
Moisture content of ODF samples. Data are presented as mean ± SD; different letters indicate significant differences (*p* < 0.05) by one-way ANOVA with Tukey’s post hoc test.

Moisture content reflects the internal pore structure and water retention capacity of ODFs [17,23]. The variation in moisture content of ODFs with different CA/β-CD inclusion complex loadings is shown in Figure 4. With the increase in the inclusion complex concentration, the moisture content of ODF samples first decreased gradually and then tended to stabilize. The control ODF exhibited the highest moisture content at 25.41%. With the addition of the CA/β-CD inclusion complex, the moisture content of the CCS-1 and CCS-2 groups decreased significantly to 16.23% and 11.57%, respectively. This behavior can be explained by the fact that the inclusion complex fills the gaps between SPI molecular chains, reducing the internal pores and free hydroxyl groups of the matrix, thereby weakening its water adsorption and retention capacity. The moisture content stabilized for the CCS-3 (9.79%) and CCS-4 (9.36%) groups, indicating that the structural compactness of the ODF matrix had reached saturation. Even with aggregate formation in the CCS-4 group, the inherent hydrophobicity of the CA in the aggregates did not enhance the water adsorption capacity of the material. This trend is consistent with the findings of Zou et al. (2021) [17], who reported that the CA/β-CD inclusion complex reduces the moisture content of polysaccharide-based composite films by enhancing the network structure and reducing free voids.

### 3.5. Surface Hydrophilicity Analysis of ODFs

**Figure 5:**
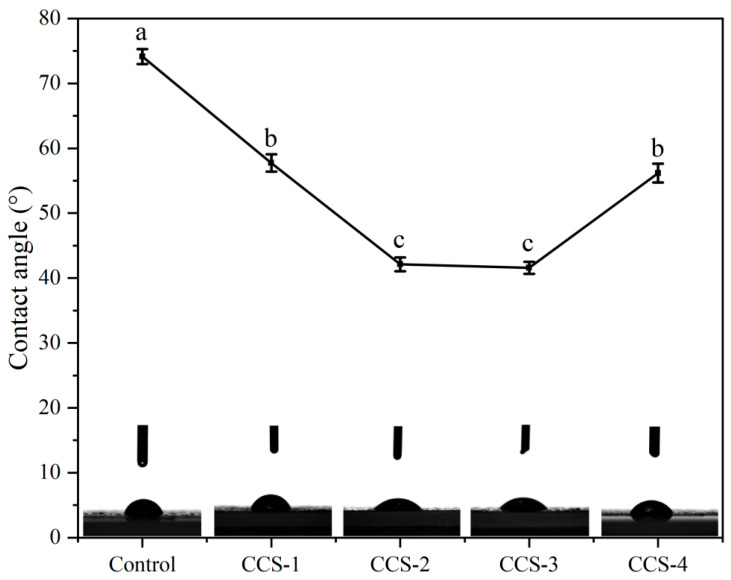
Water contact angle of ODF samples. Data are presented as mean ± SD; different letters indicate significant differences (*p* < 0.05) by one-way ANOVA with Tukey’s post hoc test.

The surface hydrophilicity of different ODFs was evaluated by measuring the water contact angle of the films. Surface hydrophilicity can reflect the affinity of ODF surface functional groups for water molecules [2]. The contact angle is less than 90° for hydrophilic ODFs and greater than 90° for hydrophobic ODFs [24]. As can be seen from Figure 5, all samples exhibited contact angles below 90°. The control ODF showed the highest contact angle at 74.13°. The contact angles of ODFs incorporated with complexes decrease significantly. This is mainly due to the abundant hydroxyl groups on the outer edge of β-CD molecules, which endow them with a strong hydrophilic tendency [25]. In the process of ODF formation, these hydrophilic groups tend to be enriched on the surface, thus significantly enhancing the hydrophilicity of the ODF surface [2,3]. From a surface thermodynamics perspective, the enrichment of hydrophilic hydroxyl groups from β-CD at the film–air interface reduces the surface free energy barrier for water spreading, thereby enhancing wettability. With increasing concentration of the inclusion complex, the contact angle generally displayed a trend of initial decrease followed by a subsequent increase. The CCS-1 and CCS-2 groups had contact angles of 57.73° and 42.12°, respectively. This indicates that within the low concentration range, the hydrophilic surface effect of the ODFs continued to strengthen as the inclusion complex concentration increased. It is worth noting that the contact angle of the CCS-3 group was 41.58°, which is equivalent to that of the CCS-2 group. This shows that the dispersion state of the inclusion complex was in balance with the exposure degree of hydrophilic groups on the surface, and no additional structure or group distribution changes occurred; therefore, the surface hydrophilicity remained relatively stable. However, upon further increasing the inclusion complex loading, the contact angle for the CCS-4 group increased to 56.17°. This turning point showed that the dominant properties of the ODF surface had changed. At this stage, the excessive amount of the inclusion complex exceeded the loading capacity of the SPI matrix, leading to agglomeration. These aggregates exposed more hydrophobic CA molecules on the ODF surface, partially offsetting the hydrophilic effect of β-CD [17]. This observation is entirely consistent with the SEM results, which showed the appearance of acicular aggregates and structural disruption on the CCS-4 surface, collectively confirming the phase separation and surface hydrophobization induced by excessive addition.

### 3.6. Surface pH and Mucoadhesiveness Analysis of ODFs

**Figure 6:**
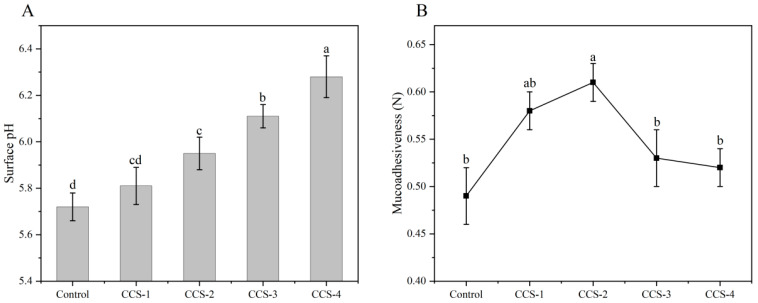
Surface pH (**A**) and mucoadhesiveness (**B**) of ODF samples. Data are presented as mean ± SD; different letters indicate significant differences (*p* < 0.05) by one-way ANOVA with Tukey’s post hoc test.

Surface pH value and oral mucoadhesiveness are key indices to evaluate the biocompatibility and delivery efficiency of ODFs [20]. An appropriate surface pH can effectively avoid mucosal irritation. Figure 6A,B present the changes in surface pH and mucoadhesive strength of ODFs, respectively. As shown in Figure 6A, all ODF samples exhibited a surface pH ranging from 5.72 to 6.28, which falls within the safe range for oral tissues and does not induce discomfort [20]. The control group had the lowest surface pH of 5.72, and the surface pH of ODFs gradually increased with increasing dosage of the inclusion complex. One possible explanation is that the hydroxyl groups of β-CD can form hydrogen bonds with the carboxyl groups of SPI, which inhibits the dissociation of the carboxyl group, and the acid sites on the ODF surface continue to decrease, making the microenvironment closer to neutrality.

Figure 6B shows that with the increase in inclusion complex content, the mucoadhesive strength of ODF first increased and then decreased. The oral adhesion of the control group was the lowest (0.49 N), while that of the CCS-2 group was the highest (0.61 N). The mucoadhesiveness of ODFs is primarily governed by physical interlocking and non-covalent molecular interactions. The uniform, continuous network structure of the CCS-2 group increases the effective contact area between the ODF and the mucin-mimetic substrate, enhancing physical interlocking between the film and the mucosal surface. Meanwhile, the abundant hydroxyl groups from β-CD and the amide groups from SPI can form extensive intermolecular hydrogen bonds with the hydroxyl and carboxyl groups in the mucin-mimetic substrate, which is the dominant molecular driving force for the enhanced mucoadhesive strength. However, excessively high loading of the inclusion complex leads to agglomeration, which destroys the continuous network structure and increases surface hydrophobicity. These changes reduce the effective contact area and the number of available hydrogen-bonding sites between the ODF and the mucosal substrate, resulting in a decrease in mucoadhesive strength for the CCS-3 and CCS-4 groups. This phenomenon is consistent with the SEM and water contact angle results and aligns with previous reports on protein-based mucoadhesive films [21].

### 3.7. Mechanical Properties Analysis of ODF

The mechanical properties, influenced by the compatibility and hydrogen-bonding interactions among ODF components, are crucial for their practical usability and reliability [26,27]. Table 1 shows the changes in TS and EAB of ODFs with different CA/β-CD inclusion complex loadings. Both the TS (6.87 MPa) and EAB (2.83%) of the control ODF were at relatively low levels. This is attributed to the aggregation of pure SPI molecular chains relying solely on weak intrinsic hydrogen bonds and hydrophobic interactions [2]. Consequently, the ODF structure was loose with insufficient intermolecular bonding, making it difficult to resist tensile stress and resulting in poor toughness. The TS and EAB showed a concomitant initial increase and subsequent decrease with increasing inclusion complex content. Optimal properties were achieved with a 10% loading (CCS-2), yielding a peak TS of 9.25 MPa and EAB of 3.56%. Compared to previously reported SPI-based films, our optimized ODF exhibits superior TS and EAB, confirming the reinforcing effect of the CA/β-CD complex. For example, Tian et al. (2024) [2] reported the maximum TS and EAB of SPI-based ODFs as 5.15 ± 0.18 MPa and 2.58 ± 0.17%, respectively. This enhancement is due to the low-dose inclusion complex filling the gaps between SPI molecular chains, while the β-CD formed additional hydrogen bonds with SPI, strengthening the intermolecular bonding force. Furthermore, the inclusion complex induced the formation of a uniformly distributed mesh structure within the SPI matrix. This structure not only reinforced the synergistic interactions between molecules, allowing the material to withstand greater tensile stress, but also accommodated more tensile deformation through the elastic deformation of the mesh [28,29]. Upon further increasing the inclusion complex concentration, both TS and EAB began to decline. The TS (8.13 MPa) and EAB (3.20%) of the CCS-3 group remained at a comparatively high level, indicating that the structural continuity of the ODF was not completely disrupted, and the intermolecular bonding force and structural toughness remained stable. In contrast, the TS of the CCS-4 group dropped sharply to 5.15 MPa, and the EAB decreased to 1.72%. This behavior can be explained by the fact that the high loading of the inclusion complex exceeded the carrying capacity of the SPI matrix, disrupting the continuous network structure and creating structural defects on the surface, as evidenced by the SEM observations.

### 3.8. Disintegration Time Analysis of ODFs

Table 1 shows the disintegration time results for the different samples. As can be seen, the disintegration time for all samples was less than 30 s, falling within the category of fast-disintegrating films [26,27]. The control ODF had the longest disintegration time at 29.31 s. With increasing concentration of the inclusion complex, the disintegration time first decreased and then increased, with the CCS-3 group showing the shortest time of 9.57 s. The accelerated disintegration upon inclusion complex addition is primarily attributed to enhanced hydrophilicity due to the abundant hydroxyl groups of β-CD, which facilitate water penetration into the film matrix [2,7]. The subsequent increase in disintegration time at 20% loading (CCS-4) is caused by inclusion complex aggregation, which reduces the effective surface area of hydrophilic groups and creates hydrophobic domains that retard water ingress [12]. Overall, the disintegration rate of ODFs was accelerated by 3.06 times after the inclusion complexes were added, which was beneficial to the release of active ingredients.

### 3.9. Thermal Stability Analysis of ODFs

**Figure 7:**
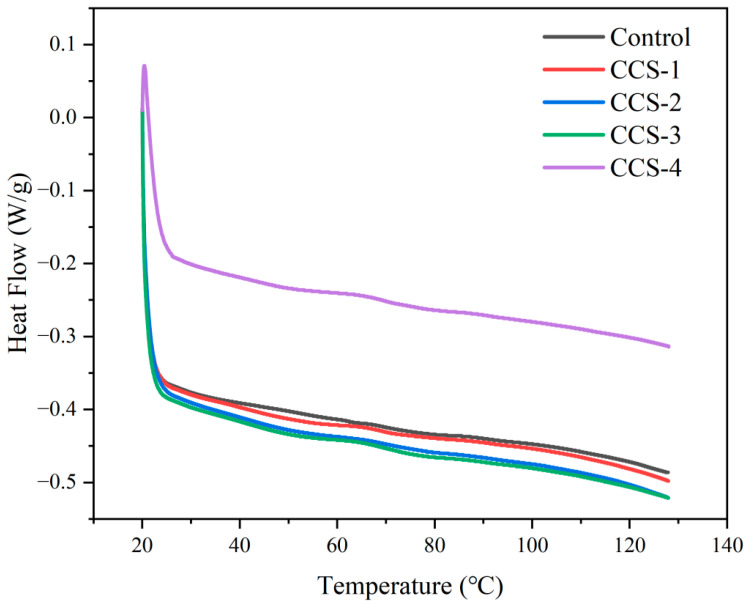
Heat flow diagram of ODF samples.

The thermal stability of different ODFs was evaluated via DSC thermograms, as shown in Figure 7. Figure S2 (Supplementary Materials) displays the Y-offset stack of the heat flow diagram for the ODF samples. The DSC thermograms of all ODF samples exhibited a broad, gradual endothermic slope from approximately 30 °C to 120 °C, without a sharp glass transition or melting peak. There is no obvious melting peak in the curve, which indicates that ODFs are typical amorphous materials [30]. The decrease in heat flow corresponds to the disruption of weak intermolecular forces and chain relaxation within the ODF components [28]. The absence of a distinct glass transition temperature (Tg) in the range of 20–120 °C suggests that the SPI matrix is in a glassy state with restricted molecular mobility, which is beneficial for maintaining structural integrity during storage. The heat flow in the control group was the highest because it takes relatively less energy to destroy the weak hydrogen bonds and hydrophobic assemblies in pure SPI. The heat flow of the membrane containing inclusion complexes (CCS-1 to CCS-3) is low, which indicates that the extra hydrogen bond between the inclusion complex and SPI forms stronger intermolecular interactions. This trend was most obvious for CCS-2 and CCS-3, which is consistent with their good continuous network structure. Notably, the heat flow for CCS-4 was higher than that of CCS-2/3, consistent with its disrupted structural homogeneity due to inclusion complex agglomeration, which weakens synergistic intermolecular forces.

To quantify the overall energy required for these processes, the enthalpy change (ΔH) was integrated over the 20–120 °C range. The calculated ΔH values are presented in Table 1. The control group had the lowest ΔH (6.2044 J/g), requiring minimal energy to initiate chain relaxation. The CCS-2 group achieved the highest ΔH (7.9496 J/g, 28.13% higher than the control), confirming that the ordered network structure (from SEM) and enhanced hydrogen bonding (from FTIR) significantly increased intermolecular interaction intensity. Excessive loading (CCS-4) reduced ΔH to 7.1238 J/g, as agglomeration disrupted the bonding network and increased chain mobility. These thermal observations are fully congruent with the structural evolution deduced from prior analyses.

### 3.10. FTIR Analysis of ODFs

**Figure 8:**
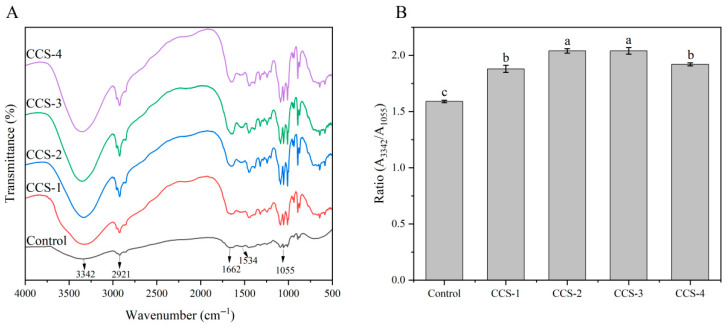
(**A**) FTIR spectra and (**B**) relative absorbance ratio (A3342/A1055) of ODF samples. Data are presented as mean ± SD; different letters indicate significant differences (*p* < 0.05) by one-way ANOVA with Tukey’s post hoc test.

Figure 8A presents the FTIR analysis of component interactions within the ODFs. The peaks at wavenumbers 2921 cm^−1^, 1662 cm^−1^, and 1534 cm^−1^ in the control group are assigned to the amide B band, amide I band, and amide II band of SPI, respectively. After the inclusion complex was added, the peak shape and absorption intensity of these characteristic bands changed significantly. This indicates that there are intermolecular interactions between SPI and the inclusion complex. The absorption band strength of the ODFs with the inclusion complex was significantly enhanced at wavenumbers of 3342 cm^−1^ and 1055 cm^−1^. These bands have been reported to be associated with hydrogen bond formation, suggesting that the introduction of the inclusion complex significantly strengthened hydrogen-bonding interactions among components within the ODF matrix [2,3,11]. Multiple studies have reported that the relative absorbance ratio of A_4000–3000_/A_1100–950_ can serve as a criterion for assessing hydrogen bond strength [2,31,32]. Figure 8B shows that the A_3342_/A_1055_ ratio was lowest for the control group and increased significantly upon incorporation of the inclusion complex. This confirms the enhancement of hydrogen-bonding interactions among components in the ODFs after the addition of the inclusion complex. Physical entanglement and non-covalent interactions, including hydrogen bonds and van der Waals forces, are postulated to be the fundamental driving forces for ODF formation [2,32]. To elucidate the interaction mechanisms among components in the ODFs, further analysis of the changes in protein secondary structure is required.

The changes in protein secondary structure of the different ODFs are shown in Table 2. Compared to the control group, the addition of the inclusion complex led to a significant increase in the contents of β-sheet and α-helix structures, alongside a significant decrease in the contents of random coil and β-turn structures. The fluctuations in the proportion of SPI secondary structures within the ODFs were highly correlated with the addition and structural role of the CA/β-CD inclusion complex. The control group showed the lowest β-sheet (29.03%) and α-helix (9.96%) contents while displaying the highest levels of random coil (18.51%) and β-turn (42.50%) among all samples. This is attributed to the aggregation of pure SPI relying solely on its weak intrinsic hydrogen bonds and hydrophobic interactions, resulting in poor conformational order of the peptide chains [33]. With increasing amounts of the inclusion complex, the proportions of β-sheet and α-helix first increased and then decreased, while the β-turn content showed an opposite trend, decreasing initially and then rising. The CCS-2 group displayed the highest proportions of β-sheet (32.83%) and α-helix (18.09%) and the lowest β-turn content (30.98%), while the random coil proportion remained relatively stable. This structural alteration can be attributed to the establishment of extra hydrogen bonds linking the inclusion complex and SPI [2]. The hydroxyl groups on the outer rim of β-CD form intermolecular hydrogen bonds with the N-H/C=O groups of the SPI peptide chains, simultaneously inducing the formation of more continuous intramolecular hydrogen bonds within the SPI chains. A dual effect of hydrogen bonds promoted the transition of peptide chains towards the ordered α-helix and β-sheet conformations, reflecting the structuring effect of the inclusion complex on the SPI secondary structure [34]. This also aligns with the continuous and regular mesh structure observed for the CCS-2 group in the prior SEM results. The proportions of β-sheet and α-helix in the CCS-3 group slightly decreased but remained at relatively high levels, indicating that the dispersion state of the inclusion complex and its hydrogen-bonding effects had reached a relative equilibrium. In contrast, the CCS-4 group showed a significant decline in β-sheet and α-helix content and a notable recovery in β-turn proportion. This is attributed to the agglomeration of the high-dose inclusion complex, which exceeds the loading capacity of the SPI matrix, disrupting the uniform hydrogen-bonding network and the orderly arrangement of peptide chains. The changes in secondary structure directly reflect the intensity of interactions between the CA/β-CD inclusion complex and SPI. Enhanced hydrogen bonding promoted an increase in the proportion of ordered structures, while the disruption of the hydrogen-bonding network caused by agglomeration led to a decrease. These findings form a logically consistent correlation with the earlier characterization results concerning ODF morphology and mechanical properties.

### 3.11. Antioxidant Capacity Analysis of ODFs

**Figure 9:**
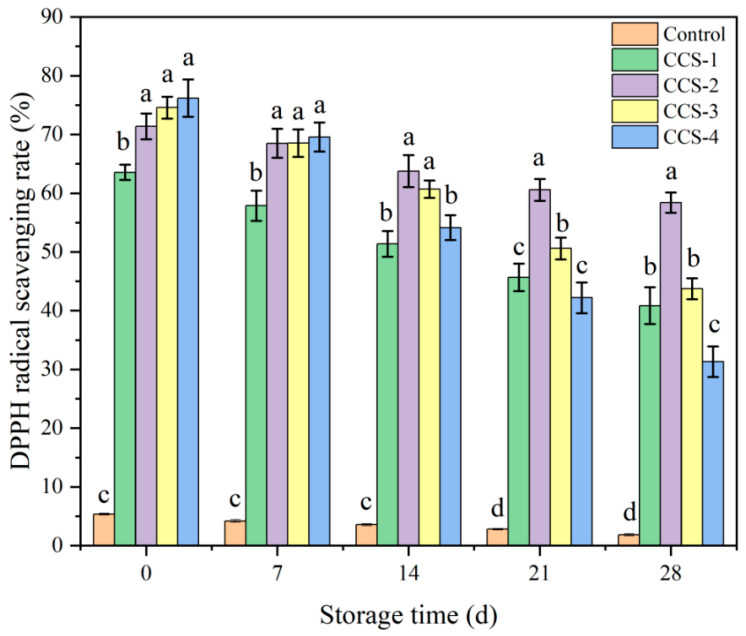
DPPH radical scavenging rate of ODF samples. Data are presented as mean ± SD; different letters indicate significant differences (*p* < 0.05) by one-way ANOVA with Tukey’s post hoc test.

The antioxidant capacity of the ODFs was evaluated by measuring the DPPH radical scavenging rate, and the results are shown in Figure 9. The control group’s DPPH radical scavenging rate was consistently lower than the other groups due to the pure SPI matrix’s limited antioxidant capacity and stability. After the inclusion complex was added, the antioxidant capacity of the ODFs was significantly improved. The DPPH radical scavenging enhancement of protein-based films through CA incorporation is also supported by findings from other research groups [35,36]. At the initial stage (0 d), the DPPH rate increased with higher loading levels of the inclusion complex. This indicates that in the initial stage, the increased loading of the active ingredient contributed to a stronger radical scavenging capability. During storage, all samples showed a gradual declining trend in their scavenging rates. With the extension of storage time, the CCS-2 group maintained a significantly higher scavenging rate than the other groups. This is due to the fact that the ordered and stable structure of the CCS-2 group reduced the oxidative degradation of the active ingredient while ensuring its sustained-release efficiency. After 28 d of storage, the scavenging rate of the CCS-2 group remained at 58.39%. In contrast, while the CCS-4 group initially showed comparable activity to CCS-2, its scavenging rate dropped sharply to 31.33% by day 28. This accelerated decay follows first-order-like kinetics, where the rate constant for antioxidant loss is significantly higher in CCS-4. The structural instability caused by inclusion complex agglomeration not only entraps part of the active ingredient, hindering its release, but also exposes CA to localized oxidative environments due to poor matrix protection, leading to rapid degradation. In contrast, the uniform network in CCS-2 provides physical protection and controlled release, thereby sustaining antioxidant activity over time. This accelerated decay results from the structural instability caused by inclusion complex agglomeration at high loading. The aggregates not only entrap part of the active ingredient, hindering its release, but also exacerbate localized autoxidation of the active substance, leading to a rapid decay in the antioxidant capacity of the ODF.

## 4. Conclusions

In conclusion, we successfully prepared an SPI-based ODF loaded with a CA/β-CD inclusion complex using the solution casting method. FTIR results show that hydrophobic interactions and hydrogen bonding are the key forces for the formation of the CA/β-CD inclusion complex. The microstructure, mechanical properties, hydrophilicity, disintegration behavior, thermal stability, and oxidation resistance are significantly improved by adding the inclusion complex to the ODFs. When the amount of inclusion complex added is 10% (*w*/*w*), the ODF forms the most orderly continuous network structure. This significantly enhances the TS, EAB, surface hydrophilicity and antioxidant capacity of the ODFs, and the disintegration speed is also significantly accelerated. However, excessive inclusion complex (20% *w*/*w*) leads to surface agglomeration of the ODFs, structural defects and performance degradation. A clear cause-and-effect relationship can be established between the micro-level interactions and the macroscopic properties of the ODFs. The enhanced intermolecular hydrogen bonding between the SPI matrix and the CA/β-CD inclusion complex induces a transition of the protein secondary structure from disordered random coils to ordered α-helices and β-sheets. This structural ordering reinforces the continuous network skeleton of the ODF, which directly translates to the significantly improved tensile strength and structural toughness. Concurrently, this dense and ordered network effectively restricts oxygen permeation and encapsulates the active compounds, thereby minimizing degradation kinetics and resulting in excellent sustained antioxidant stability. To further enhance the impact of this work, future studies could explore in vivo performance, sensory evaluation, and scalability of the optimized formulation, as well as the applicability of this platform to other hydrophobic bioactive compounds.

## Figures and Tables

**Table 1:** Mechanical properties, disintegration times, and ΔH results of the ODF samples.

Samples	TS (MPa)	EAB (%)	Disintegration Time (s)	ΔH (J/g)
Control	6.87 ± 0.29 ^c^	2.83 ± 0.07 ^c^	29.31 ± 1.34 ^a^	6.20 ± 0.13 ^c^
CCS-1	8.18 ± 0.31 ^b^	3.21 ± 0.11 ^b^	16.29 ± 1.67 ^b^	7.29 ± 0.13 ^b^
CCS-2	9.25 ± 0.31 ^a^	3.56 ± 0.10 ^a^	10.38 ± 0.74 ^c^	7.95 ± 0.11 ^a^
CCS-3	8.13 ± 0.37 ^b^	3.20 ± 0.10 ^b^	9.57 ± 0.91 ^c^	7.92 ± 0.12 ^a^
CCS-4	5.15 ± 0.29 ^d^	1.72 ± 0.11 ^d^	14.86 ± 0.75 ^b^	7.12 ± 0.17 ^b^

Note: Data are presented as mean ± SD; different letters indicate significant differences (*p* < 0.05) by one-way ANOVA with Tukey’s post hoc test.

**Table 2:** Results of secondary structure changes in ODF samples.

Samples	β-Sheet (%)	Random Coil (%)	α-Helix (%)	β-Turn (%)
Control	29.03 ± 0.56 ^d^	18.51 ± 0.11 ^a^	9.96 ± 0.45 ^c^	42.50 ± 0.30 ^a^
CCS-1	31.30 ± 0.27 ^b^	18.16 ± 0.08 ^b^	17.19 ± 0.36 ^b^	33.35 ± 0.33 ^c^
CCS-2	32.83 ± 0.33 ^a^	18.11 ± 0.11 ^b^	18.09 ± 0.21 ^a^	30.98 ± 0.36 ^e^
CCS-3	32.48 ± 0.31 ^a^	18.17 ± 0.13 ^b^	17.08 ± 0.37 ^b^	32.27 ± 0.43 ^d^
CCS-4	30.56 ± 0.25 ^c^	18.27 ± 0.12 ^b^	16.79 ± 0.31 ^b^	34.38 ± 0.29 ^b^

Note: Data are presented as mean ± SD; different letters indicate significant differences (*p* < 0.05) by one-way ANOVA with Tukey’s post hoc test.

## Data Availability

The original contributions presented in this study are included in the article/Supplementary Materials. Further inquiries can be directed to the corresponding authors.

## Supplementary Materials

The following supporting information can be downloaded at https://www.mdpi.com/article/10.3390/foods15081410/s1, Figure S1: The chemical structures of CA and β-CD; Figure S2: Y-offset stack of the heat flow diagram for the ODF samples.

## References

[1] Eliphaz M. (2016). Formulation of a Natural Intraoral Dispersible Film (IDF) for Intraoral Delivery of Various Natural Drugs Using Edible Rice Paper Film as the Carrier Vehicle.

[2] Tian Y., Liu C., Cheng J., Li J., Wang Z., Yuan C., Zhou L. (2024). Development of soy protein isolate-based orally disintegrating film: Improvement of disintegration speed, mechanical properties, and thermal stability by β-Cyclodextrin. LWT.

[3] Sha H., Yuan C., Cui B., Zhao M., Wang J. (2022). Pre-gelatinized cassava starch orally disintegrating films: Influence of β-Cyclodextrin. Food Hydrocoll..

[4] Wang Q., Chen W., Zhu W., McClements D.J., Liu X., Liu F. (2022). A review of multilayer and composite films and coatings for active biodegradable packaging. npj Sci. Food.

[5] Remedio L.N., dos Santos Garcia V.A., Rochetti A.L., Berretta A.A., Yoshida C.M.P., Fukumasu H., Vanin F.M., de Carvalho R.A. (2023). Hydroxypropyl methylcellulose orally disintegration films produced by tape casting with the incorporation of green propolis ethanolic extract using the printing technique. Food Hydrocoll..

[6] Costa B.S., Teixeira C.T.G.V.M., Chambi H.N., Schmidt F.L. (2025). Pectin orally disintegrating films containing jambolan juice (Syzygium cumini) and ginger: Functional properties. Ind. Crops Prod..

[7] Khatreja K., Santhiya D. (2024). Physicochemical characterization of novel okra mucilage/hyaluronic acid-based oral disintegrating films for functional food applications. Int. J. Biol. Macromol..

[8] Mathai J.K. (2018). Digestible Indispensable Amino Acid Scores for Food Proteins. Ph.D. Thesis.

[9] Chen L., Ramezan Y., Pourramezan H., Najafi A., Kamkari A., Goksen G., Huang Z., Zhang W. (2025). Soy Protein Isolate (SPI)-Based Films/Coatings for Food Packaging: Research Progress on Properties and Applications. Compr. Rev. Food Sci. Food Saf..

[10] Wan Z.-L., Guo J., Yang X.-Q. (2015). Plant protein-based delivery systems for bioactive ingredients in foods. Food Funct..

[11] Jiang L., Jia F., Han Y., Meng X., Xiao Y., Bai S. (2021). Development and characterization of zein edible films incorporated with catechin/β-cyclodextrin inclusion complex nanoparticles. Carbohydr. Polym..

[12] Sha H., Cui B., Yuan C., Li Y., Guo L., Liu P., Wu Z. (2022). Catechin/β-cyclodextrin complex modulates physicochemical properties of pre-gelatinized starch-based orally disintegrating films. Int. J. Biol. Macromol..

[13] Tian Y., Yuan C., Cui B., Lu L., Zhao M., Liu P., Wu Z., Li J. (2022). Pickering emulsions stabilized by β-cyclodextrin and cinnamaldehyde essential oil/β-cyclodextrin composite: A comparison study. Food Chem..

[14] Ou Y., Yan M., Gao G., Wang W., Lu Q., Chen J. (2022). Cinnamaldehyde protects against ligature-induced periodontitis through the inhibition of microbial accumulation and inflammatory responses of host immune cells. Food Funct..

[15] de Oliveira I.C., Galvão-Moreira L.V., Vilela J.L., Duarte-Silva M., Aguiar-da-Silva L.D., Pereira C.A., Pereira D.M., Pinheiro A.J., Lima-Neto L.G., Fernandes E.S. (2023). Cinnamaldehyde modulates host immunoinflammatory responses in rat ligature-induced periodontitis and peripheral blood mononuclear cell models. Int. Immunopharmacol..

[16] Varghese S.A., Siengchin S., Parameswaranpillai J. (2020). Essential oils as antimicrobial agents in biopolymer-based food packaging-A comprehensive review. Food Biosci..

[17] Zou Y., Yuan C., Cui B., Wang J., Yu B., Guo L., Dong D. (2021). Mechanical and antimicrobial properties of high amylose corn starch/konjac glucomannan composite film enhanced by cinnamaldehyde/β-cyclodextrin complex. Ind. Crops Prod..

[18] Ma J., Fan J., Xia Y., Kou X., Ke Q., Zhao Y. (2023). Preparation of aromatic β-cyclodextrin nano/microcapsules and corresponding aromatic textiles: A review. Carbohydr. Polym..

[19] Suvarna V., Chippa S. (2023). Current overview of cyclodextrin inclusion complexes of volatile oils and their constituents. Curr. Drug Deliv..

[20] dos Santos Garcia V.A., Borges J.G., Osiro D., Vanin F.M., de Carvalho R.A. (2020). Orally disintegrating films based on gelatin and pregelatinized starch: New carriers of active compounds from acerola. Food Hydrocoll..

[21] Alaei S., Omidi Y., Omidian H. (2021). In vitro evaluation of adhesion and mechanical properties of oral thin films. Eur. J. Pharm. Sci..

[22] Zou Y., Yuan C., Cui B., Sha H., Liu P., Lu L., Wu Z. (2021). High-amylose corn starch/konjac glucomannan composite film: Reinforced by incorporating β-cyclodextrin. J. Agric. Food Chem..

[23] Li J., Ye F., Lei L., Zhao G. (2018). Combined effects of octenylsuccination and oregano essential oil on sweet potato starch films with an emphasis on water resistance. Int. J. Biol. Macromol..

[24] Ma Y., Cao X., Feng X., Ma Y., Zou H. (2007). Fabrication of super-hydrophobic film from PMMA with intrinsic water contact angle below 90. Polymer.

[25] Liu T., Li J., Lei H., Zhen X., Wang Y., Gou D., Zhao J. (2023). Preparation of chitosan/β-cyclodextrin composite membrane and its adsorption mechanism for proteins. Molecules.

[26] Li Y., Yuan C., Cui B., Liu P., Fang Y., Wu Z., Zhao H., Liu J. (2025). Chitosan/pre-gelatinized waxy corn starch composite edible orally disintegrating film for taurine delivery. Food Hydrocoll..

[27] Singh P., Kaur G., Singh A., Sharma T., Dar B. (2023). Improved mechanical, functional and antimicrobial properties of corn starch-based biodegradable nanocomposites films reinforced with lemongrass oil nanoemulsion and starch nano-crystal. Mater. Chem. Phys..

[28] Zhao Y., Shi L., Ren Z., Liu Q., Zhang Y., Weng W. (2024). Physicochemical and antimicrobial properties of soy protein isolate films incorporating high internal phase emulsion loaded with thymol. Food Chem. X.

[29] Wang Y.-L., Li R., Yang X., Li L. (2025). Multifunctional film of soy protein nanofiber/chitosan/zinc oxide for tomato postharvest freshness preservation. Food Hydrocoll..

[30] Sahoo D., Priyadarshini P., Dandela R., Alagarasan D., Ganesan R., Varadharajaperumal S., Naik R. (2021). Investigation of amorphous-crystalline transformation induced optical and electronic properties change in annealed As50Se50 thin films. Opt. Quantum Electron..

[31] Oh S.Y., Yoo D.I., Shin Y., Seo G. (2005). FTIR analysis of cellulose treated with sodium hydroxide and carbon dioxide. Carbohydr. Res..

[32] Xiao Y., Liu Y., Kang S., Wang K., Xu H. (2020). Development and evaluation of soy protein isolate-based antibacterial nanocomposite films containing cellulose nanocrystals and zinc oxide nanoparticles. Food Hydrocoll..

[33] Hu X., Zeng Z., Zhang J., Wu D., Li H., Geng F. (2023). Molecular dynamics simulation of the interaction of food proteins with small molecules. Food Chem..

[34] Fu H., Li J., Yang X., Swallah M.S., Gong H., Ji L., Meng X., Lyu B., Yu H. (2023). The heated-induced gelation of soy protein isolate at subunit level: Exploring the impacts of α and α′ subunits on SPI gelation based on natural hybrid breeding varieties. Food Hydrocoll..

[35] Zhao X., Mu Y., Dong H., Zhang H., Zhang H., Chi Y., Song G., Li H., Wang L. (2021). Effect of cinnamaldehyde incorporation on the structural and physical properties, functional activity of soy protein isolate-egg white composite edible films. J. Food Process. Preserv..

[36] Yin W., Yan R., Zhou X., Li X., Sang S., McClements D.J., Chen L., Long J., Jiao A., Wang J. (2023). Preparation of robust, water-resistant, antibacterial, and antioxidant chitosan-based films by incorporation of cinnamaldehyde–tannin acid-zinc acetate nanoparticles. Food Chem..

