# Supplementary material for: Cinnamaldehyde/β-Cyclodextrin Inclusion Complex Enhances Physicochemical and Antioxidant Properties of Edible Orally Disintegrating Film
Source: Foods. 2026 Apr 17;15(8):1410. doi: 10.3390/foods15081410 (PMC13114787; doi:10.3390/foods15081410)
Supplement: Supplementary file 1 [file foods-15-01410-s001.zip › foods-4227080-supplementary.pdf]

CA

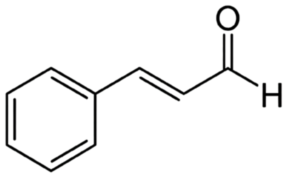

β-CD

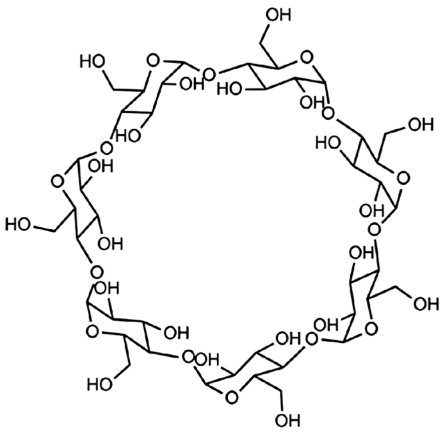

Figure S1. Chemical structures of CA and β-CD

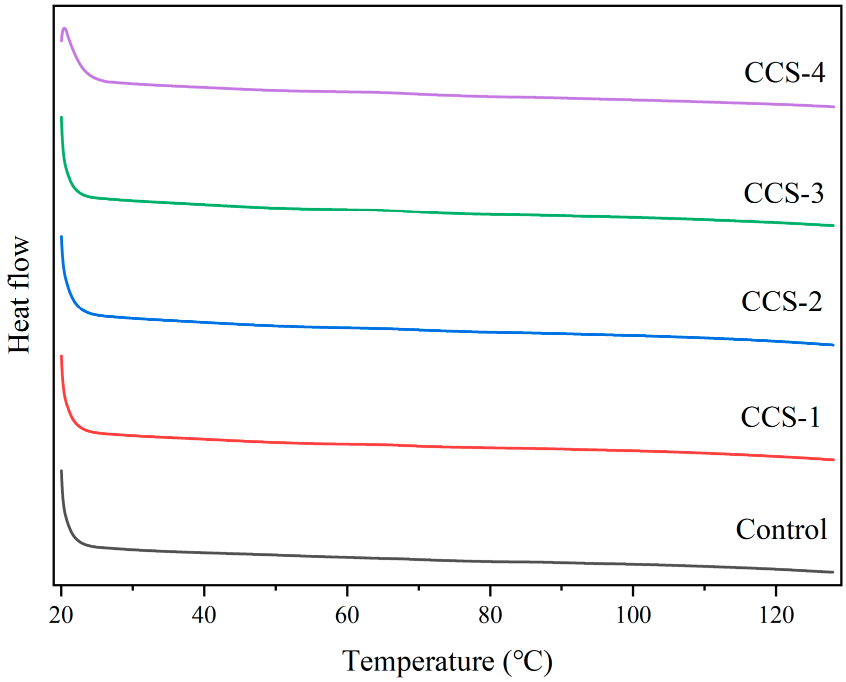

Figure S2. Heat flow diagram of ODF samples(Y-Offset Stacked)
